# Relationship between the temporal course of astrogliosis and symptom improvement in cerebral infarction: report of a case monitored using ^18^F-THK5351 positron emission tomography

**DOI:** 10.1186/s12880-020-00481-4

**Published:** 2020-07-14

**Authors:** Kenji Ishibashi, Yoshiharu Miura, Kosei Hirata, Jun Toyohara, Kenji Ishii

**Affiliations:** 1 Research Team for Neuroimaging, Tokyo Metropolitan Institute of Gerontology, 35-2 Sakae-cho, Itabashi-ku, Tokyo, 173-0015 Japan; 2 Department of Neurology, Tokyo Metropolitan Cancer and Infectious Diseases Center Komagome Hospital, Tokyo, Japan; 3 Department of Neurology and Neurological Science, Tokyo Medical and Dental University, Tokyo, Japan

**Keywords:** ^18^F-THK5351, Positron emission tomography, Cerebral infarction, Astrogliosis, Case report

## Abstract

**Background:**

^18^F-THK5351 was recently shown to bind to monoamine oxidase B (MAO-B) with high affinity. MAO-B is highly concentrated in astrocytes and increases during astrogliosis. Therefore, ^18^F-THK5351 accumulates in lesions undergoing astrogliosis. Cerebral infarction causes astrogliosis, which may be beneficial for repairing and regenerating injured cells and tissues in the lesions. Therefore, monitoring the degree of astrogliosis and stroke symptoms is essential for understanding the roles of astrogliosis in cerebral infarction.

**Case presentation:**

A 72-year-old man, complaining of total loss of sensation in the left index finger, was diagnosed with acute cerebral infarction, and underwent ^18^F-THK5351 positron emission tomography (PET) on two occasions after the stroke. The first PET scan performed on day 27 revealed intense uptake in the infarct lesion located around the right precentral and postcentral gyri. However, the second PET scan on day 391 showed that the uptake had diminished significantly. The sensory deficit in the left index finger had improved by 30 and 70% at the times of the first and second PET scans, respectively.

**Conclusions:**

^18^F-THK5351 uptake in the infarct lesion evidently changed between days 27 and 391, along with improved sensory deficit in the left index finger. Astrocytes reportedly play a role in restoring neuronal integrity. Therefore, the temporal course of astrogliosis may have been related to improving stroke symptoms in this patient, suggesting that the degree of astrogliosis in the infarct lesion may aid in assessing the prognosis in stroke patients.

## Background

The positron emission tomography (PET) radioligand, ^18^F-THK5351, has been shown to bind monoamine oxidase B (MAO-B) with high affinity [1–3]. Representative ^18^F-THK5351 uptake sites in normal conditions are observed in the striatum, thalamus, limbic system, and brainstem, and this distribution pattern is consistent with the established distribution pattern of MAO-B [4, 5]. More recently, postmortem examination in a glioblastoma case demonstrated that intense uptake in antemortem ^18^F-THK5351 PET images is reflective of MAO-B concentrations in the glioblastoma lesion [6]. Since ^18^F-THK5351 was initially developed for targeting tau aggregates in neurofibrillary tangles [7, 8], it is now recognized as a dual-purpose compound that binds to both MAO-B and tau aggregates. In cases in which tau pathology is irrelevant, such as those in young subjects or those involving regions where tau protein does not usually accumulate, ^18^F-THK5351 uptake is considered to reflect MAO-B concentration.

MAO-B is highly concentrated in astrocytes and serotonergic and histaminergic neurons [4]. However, astrocytes can easily proliferate in response to inflammation caused by brain injury and diseases [9]. Therefore, regional changes in MAO-B concentration can be an index of astrocyte reactivity, known as “astrogliosis”. These findings suggest that ^18^F-THK5351 accumulates in lesions where astrogliosis occurs, and that ^18^F-THK5351 PET can be a powerful biomarker for visualizing and quantifying astrogliosis, as recently demonstrated in patients with neurological disorders [10–13]. More importantly, longitudinal assessment of the degree of astrogliosis in the living human brain can provide new insights into the pathophysiology of neurological disorders.

Here, we report a case of cerebral infarction with an unusual symptom, a sensory deficit in the left index finger, in which the degree of astrogliosis and stroke symptoms was monitored. Although a small number of reports have described stroke-related changes on ^18^F-THK5351 PET in a cross-sectional manner [10–12], we believe this is the first report that describes the time course of ^18^F-THK5351 uptake in a patient with cerebral infarction. We then discuss the possible role of astrogliosis in the infarct lesion, focusing on the relationship between the temporal course of astrogliosis and symptom improvement.

## Case presentation

The patient was a 72-year-old man without significant medical history, except for antihypertensive treatment over the last several years. After suddenly developing a sensory disturbance in the left index finger he attended the Department of Neurology at the Tokyo Metropolitan Cancer and Infectious Diseases Center Komagome Hospital. Neurological examination showed a complete loss of pain, temperature, and touch sensations in the distal part of the left index finger, without disturbances in motor function. Electrocardiography showed atrial fibrillation, and diffusion-weighted magnetic resonance imaging (MRI) revealed a high-intensity lesion located around the right precentral and postcentral gyri. The patient was diagnosed with acute cerebral infarction.

After giving written informed consent, the patient underwent ^18^F-THK5351 PET, concurrently with follow-up MRI examinations. These examinations were performed 27 days and 391 days after the stroke to monitor the degree of astrogliosis in the infarct lesion. Oral anticoagulant therapy (apixaban) was started after the stroke, and no reinfarction had occurred by the time of the second PET scan. Follow-up neurological examinations showed that the sensory disturbance in the left index finger had improved by 30 and 70% at the times of the first and second ^18^F-THK5351 PET examinations, respectively.

^18^F-THK5351 PET examinations were performed at the Tokyo Metropolitan Institute of Gerontology using the Discovery PET/CT 710 scanner (GE Healthcare, Milwaukee, WI, USA) in three-dimensional mode. Emission data were acquired for 20 min, starting 40 min after intravenous injection of ^18^F-THK5351 at a dose of approximately 190 MBq (5.1 mCi). Images with 47 slices were obtained with a voxel size of 2 × 2 × 3.27 mm and a matrix size of 128 × 128. The data were reconstructed after correcting for decay, attenuation, and scatter. ^18^F-THK5351 PET images were then normalized using the cerebellum as a reference region, where cerebellar uptake was set as one.

**Fig. 1:**
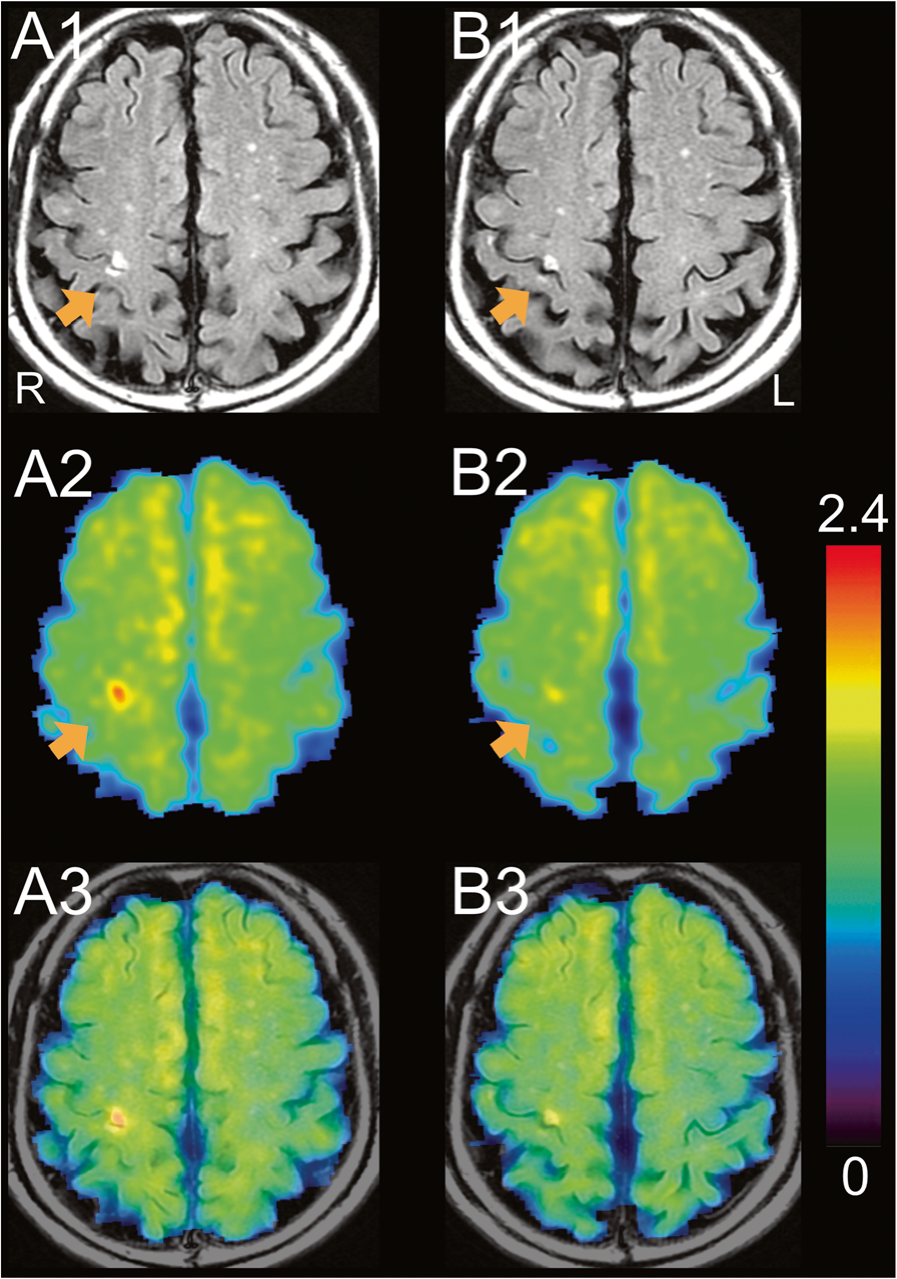
FLAIR and ^18^F-THK5351 PET images, 27 days (**A**) and 391 days (**B**) after stroke. FLAIR (**A1**, **B1**) and ^18^F-THK5351 PET (**A2**, **B2**) images are displayed in the upper and middle rows, respectively. The orange arrows represent the infarct lesion located around the right precentral and postcentral gyri. The fused images of FLAIR and ^18^F-THK5351 PET are displayed in the bottom row (**A3**, **B3**). The rainbow color scale represents the normalized uptake of ^18^F-THK5351, where cerebellar uptake was set as one

Fluid-attenuated inversion recovery (FLAIR) images, 27 days and 391 days after the stroke, are shown in Fig. 1A1 and B1, respectively. The infarct lesion was identified as the high intensity located around the right precentral and postcentral gyri (orange arrows). Even after more than 1 year had passed since the stroke, the high intensity remained in FLAIR images (Fig. 1B1). The first and second ^18^F-THK5351 PET images were co-registered to each of the corresponding FLAIR images, and are shown in Fig. 1A2 and B2, respectively. ^18^F-THK5351 PET images superimposed on FLAIR images, 27 days and 391 days after the stroke, are shown in Fig. 1A3 and B3, respectively. On day 27, ^18^F-THK5351 PET image revealed intense uptake (arrow in Fig. 1A2), and the fused image (Fig. 1A3) confirmed that the lesion was anatomically identical in FLAIR and ^18^F-THK5351 PET images. On day 391, ^18^F-THK5351 PET images showed significantly reduced uptake in the infarct lesion (arrow in Fig. 1B2).

## Discussion and conclusion

We present the temporal course of astrogliosis in an infarct lesion, using ^18^F-THK5351 PET. Recent studies in the living human brain have shown considerable reductions in ^18^F-THK5351 uptake after administration of MAO-B inhibitors, selegiline or rasagiline [1, 3]. MAO-B is predominantly expressed on the outer mitochondrial membrane of astrocytes [4]. In a study comparing antemortem ^18^F-THK5351 PET images and postmortem pathological specimens, regional MAO-B concentrations were positively correlated with in vivo ^18^F-THK5351 uptake and in vitro glial fibrillary acidic protein level, which is an established marker for astrocyte activation and astrogliosis [2]. These findings suggest that ^18^F-THK5351 uptake is associated with the degree of astrogliosis. Although ^18^F-THK5351 also binds to tau aggregates [7, 8], tau pathology is usually not relevant to the infarct lesion. Therefore, temporally changed ^18^F-THK5351 uptake in this case is likely associated with temporally changed MAO-B concentrations, which are reflective of changes in the degree of astrogliosis.

Histopathological findings caused by cerebral infarction can be ordered chronologically, as follows: acute neuronal injury (up to a few days), acute inflammation (up to a few weeks), chronic inflammation (up to decades), and resorption (up to decades) [14–16]. Astrocytes begin to proliferate and migrate during the acute inflammation phase. The response gradually transitions into the chronic inflammation phase, where astrocytes play a pivotal role in scar formation. The resorption phase is characterized by the absence of an inflammatory response; thus, astrogliosis is observed especially in the acute and chronic inflammatory phases. This established knowledge is based on the results from cross-sectional studies of postmortem brains, and longitudinal observations of the degree of astrogliosis in an individual infarct lesion has not been reported previously. Thus, this is the first description of the evolution of astrogliosis in a living patient with cerebral infarction, and may provide new insight into the role of astrogliosis in stroke.

In this case, ^18^F-THK5351 uptake changed significantly between the first and second PET images, and the sensory deficit in the left index finger had improved by 30 and 70% at the times of the first and second PET scans, respectively. However, the FLAIR images were almost unchanged. Thus, the type of information is qualitatively different between ^18^F-THK5351 PET and FLAIR images. Since ^18^F-THK5351 PET was performed 27 days and 391 days after the stroke, the changes in ^18^F-THK5351 uptake likely represent the temporal course of astrogliosis from chronic inflammation to resorption phases in the infarct lesion. Astrocytes play multiple roles throughout the course of astrogliosis and exert numerous beneficial effects, which protect cells and tissues. Astrogliosis in the infarct lesion may facilitate restoration of neuronal integrity by producing growth factors involved in repair and regenerative neuronal mechanisms [16, 17]. These findings suggest that the temporal course of astrogliosis, observed as changes in ^18^F-THK5351 uptake, may have been related to the improvement in this patient’s sensory deficit, and that assessment of the degree of astrogliosis in the infarct lesion may predict the possibility of improvement in motor and sensory disabilities. For example, persistently intense ^18^F-THK5351 uptake may suggest sustained improvement of disability. On the other hand, diminished ^18^F-THK5351 uptake may suggest absence of further recovery. Studies with a large number of subjects are required to confirm this hypothesis.

In conclusion, we report a case of cerebral infarction in which the degree of astrogliosis and stroke symptoms was monitored. ^18^F-THK5351 uptake in the infarct lesion changed dynamically between days 27 and 391, along with improved sensory deficit in the left index finger. Since astrocytes reportedly play a role in restoring neuronal integrity, the temporal course of astrogliosis may have been related to improving stroke symptoms in this patient. This case suggests that the assessment of the degree of astrogliosis in the infarct lesion may predict the possibility of improvement in stroke symptoms.

## Data Availability

The datasets used and/or analyzed during the current study are available from the corresponding author on reasonable request.

## Abbreviations

PET: Positron emission tomography
MAO-B: Monoamine oxidase B
MRI: Magnetic resonance imaging
FLAIR: Fluid-attenuated inversion recovery
